# Congenital lower lip pits: Van der Woude syndrome

**DOI:** 10.4317/jced.54953

**Published:** 2018-11-01

**Authors:** Veenu Gurpal-Chhabda, Gurpal Singh-Chhabda

**Affiliations:** 1 3rd year MDS student, Department of Oral Medicine and Radiodiagnosis, Maitri Dental College and research, Anjora, Durg, C.G.; 2 Consultant Plastic and Reconstructive Surgery, Shri Balaji Superspeciality Hospital, Raipur, C.G.

## Abstract

The Van der Woude syndrome is a rare autosomal dominant development malformation characterized by a paramedian lip pits and /or sinuses or conical elevation of lower lip associated with cleft lip and or palate. These congenital lip pits usually appear clinically in the vermilion border of lip, with or without secretion. The critical region of VWS has been identified to be at Iq32 to 41 with high, but incomplete penetrance and variable expressivity. Therapeutic intervention is generally required for cosmetic reason or when recurrent inflammation is present. Dental surgeon should be aware of this syndrome, as it is associated with variety of other congenital malformation. van der woude syndrome can be easily missed if it is not in the back of mind and its associated congenital malformation if present. We report a case of lower lip pits with bilateral cleft lip.

** Key words:**Van der Woude Syndrome, congenital pits, cleft lip / palate.

## Introduction

Van der Woude syndrome (VWS) also known as lip pit syndrome is a rare autosomal dominant disorder with incidence of about 1 in 75,000 to 1 in 100,000 and without gender predilection (1). Demarquay (2) was the first to describe congenital lip sinuses (1845). Van der Woude (1954) (3) identified the association between lower lip sinuses and cleft lip and palate. VWS syndrome is the most common cleft syndrome occurring in about 2% of population with facial cleft (4). Genetic defect of lip pit was found to be due to micro deletion on chromosome bands Iq32-q4 (5,6). More recently a mutation in the IRF6gene was identified (7). The main clinical manifestations are pits and/or sinuses of the lower lip associated with cleft lip and /or palate and occasionally hypodontia (8). Congenital lip pits manifest clinically in the vermilion border of the lip, with or without discharge. They are usually bilateral but may be unilateral or localized centrally on the lower lip (9). Lower lip pits have been described to be associated with other congenital anomalies (10,11). The indication for surgical treatment of congenital lip sinuses is primarily cosmetic, although recurrent inflammation is also considered (12).

We present a case of Van der woude syndrome that was already operated for bilateral cleft lip in childhood and now presented with lower lip pits which could have been missed if the upper lip scar of operated cleft lip would not have been there.

## Case Report

**Figure 1:**
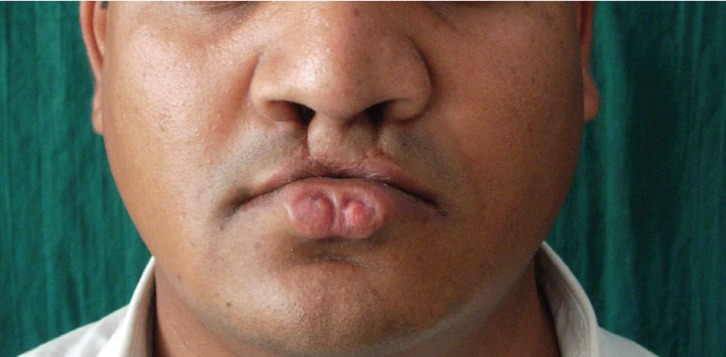
Preoperative photograph showing lower lip pits bilaterally in the midline.

**Figure 2:**
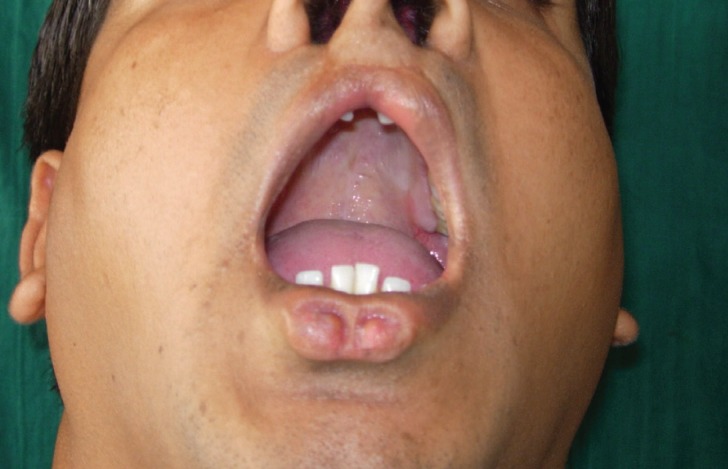
Preoperative photographs with open mouth showing no features of cleft palate.

A 2O year old male presented with two-rounded depression and pronounced lower lip along with a scar mark in the upper lip at both sides of midline (Fig. 1). He was operated for bilateral cleft lip in his childhood when he was four years old. On examination bilateral symmetric pits were seen in the vermilion border of the lower lip separated by about 3mm (Fig. 1). These pits were present since birth as per the patient. The pit on the right side had depth of 3mm and that on the left had 4mm. There was no discharge from the pit either spontaneously or on pressure. Teeth’s of maxillary and mandibular arch were normal. Other pathologic condition such as hypodontia, supernumerary tooth, syngnathia, symblepharon and cleft palate were not found on clinical examination (Fig. 2). Dye study was not performed, as clinically there was no discharge from the pit.

The above findings and the association with cleft lip led us to the diagnosis of Van der Woude syndrome .Our case was probably the new mutation as there was no similar history in the family.

**Figure 3:**
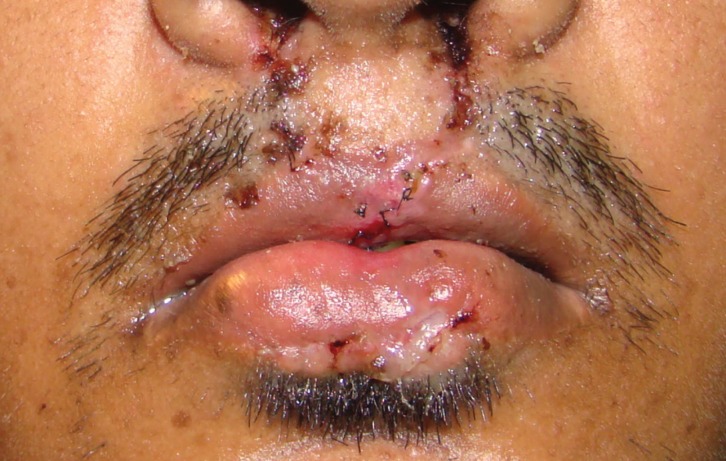
Postoperative photograph showing scar mark over lower lip.

Surgery was done for the cosmetic reason as demanded by patient for both the lips in Balaji Suoerspeciality Hospital, Raipur by plastic surgeon . Lower lip sinus was excised in toto. No communicating tracts with salivary glands were found. For the upper lip, revision of the scar was done. Patient after 10 days of follow up (Fig. 3) in our outpatient department (OPD) had no mucocoele formation or discharge from the site. Informed consent has been obtained by the patient.

## Discussion

Van der Woude syndrome is a rare autosomal dominant development malformation characterized by lower lip pits, Cleft lip, Cleft palate or both and occasionally hypodontia (3,8). Incidence of Van der Woude syndrome has been reported to be 1 in 75,000 to 1 in 100,000 live births (1). It is the most common form of syndromic clefting. Congenital lower lip pits accounts for 2% of all cases of clefting (4). The mode of inheritance is autosomal dominant with 80% to 100% penetrance, but with variable clinical expression. The gene responsible for V.W.S. has been mapped to the long arm of chromosome 1 at q32 to q41 (VWS1). Second VWS locus (VWS2) has been mapped to Ip34. Kondo *et al.* (7) identified mutation in the gene encoding interferon regulatory factor-6 (IRF-6). Mutation in IRF-6 can also cause Popliteal pterygium syndrome which shows some features in common with VWS.

Etiology of lower lip pits is considered to be due to failure of obliteration of the lateral sulci of the developing mandibular arch. This may also be due to lack of fusion of the lower part of the first branchial arch. This results in cleft of other structures coming from the lower part of the first branchial arch. (7) 

The main feature of VWS is lip pits. These pits are depressions of the lower lip that represent blind sinuses or fistulas that may extend deep into the orbicularis muscle (3). Sometimes these pits may communicate with the underlying minor salivary gland thereby discharging saliva. These pits are situated usually on the border between vermilion and mucosa. The depth of these pits is between 5mm to 25mm. (13). They usually occur on either side of the midline of the lower lip (Fig. 1) and are generally bilateral (14). Clinically these pits appear as asymptomatic (our case) with only small depression on the vermilion border or fistula that penetrates into the adjacent minor salivary gland discharging saliva.

Congenital lip pits may be associated with other developmental anomalies. They have been noted in association with syngnathia, hypodontia,symblepheron, syndactyly, polythelia, bilateral talipes equinovarus , mental retardation, ankyloblepharon, uvula bifida, chronic otitis media and anomalies of the extremities, sternum or heart (10,11). Other syndromes which are having lower lip pits as characteristic feature include oral facial digital syndrome type 1, popliteal pteryrigium syndrome and kabuki make up syndrome (15-17). Oral facial digital syndrome represents group of congenital anomalies affecting face, oral structures and digits. This condition was divided into 9 syndromes with OFD1 being the most common. OFD1 is an x chromosome linked dominant trait mostly affecting females. Popliteal pterygium syndrome is a rare autosomal disorder with having same features as that of orofacial syndrome along with skin and genital anomalies. Kabuki make up syndrome features include dysmorphic face, post-natal growth retardation, skeletal abnormalities, mental retardation and unusual dermatoglyphic patterns.

Indications for surgical intervention of congenital lip sinus are treatment of the associated cosmetic deformity and recurrent inflammation. (12). There are certain difficulties observed in complete excision of the sinus. Recurrent mucocoele formation is common complication noted following excision. Thus during surgery importance of meticulous removal of all minor salivary glands draining into the tracts has been stressed.

## Conclusions

Dental surgeons should be aware of this rare syndrome, as it is associated with variety of other congenital and development anomalies. Genetic counseling is needed and meticulous excision of the pits is required.
